# Cascade synthesis of uridine-5′-diphosphate glucuronic acid by coupling multiple whole cells expressing hyperthermophilic enzymes

**DOI:** 10.1186/s12934-019-1168-z

**Published:** 2019-07-01

**Authors:** Dan-Hua Meng, Ran-Ran Du, Lu-Zhou Chen, Meng-Ting Li, Fei Liu, Jin Hou, Yi-Kang Shi, Feng-Shan Wang, Ju-Zheng Sheng

**Affiliations:** 1 Key Laboratory of Chemical Biology of Natural Products (Ministry of Education), School of Pharmaceutical Sciences, Shandong University, Jinan, 250012 China; 2 Key Laboratory of Biopharmaceuticals, Shandong Academy of Pharmaceutical Sciences, Jinan, 250101 China; 3 State Key Laboratory of Microbiology, Shandong University, Jinan, 250100 China; 4 National Glycoengineering Research Center, Shandong University, Jinan, 250012 China

**Keywords:** Biocatalysis, Whole cell synthesis, Nucleotide sugar, UDP-GlcA, Hyperthermophilic enzyme, NAD^+^ regeneration

## Abstract

**Background:**

Enzymatic glycan synthesis has leapt forward in recent years and a number of glucuronosyltransferase (EC 2.4.1.17) have been identified and prepared, which provides a guide to an efficient approach to prepare glycans containing glucuronic acid (GlcA) residues. The uridine 5′-diphosphate (UDP) activated form, UDP-GlcA, is the monosaccharide donor for these glucuronidation reactions.

**Results:**

To produce UDP-GlcA in a cost-effective way, an efficient three-step cascade route was developed using whole cells expressing hyperthermophilic enzymes to afford UDP-GlcA from starch. By coupling a coenzyme regeneration system with an appropriate expression level with UDP-glucose 6-dehydrogenase in a single strain, the cells were able to meet NAD^+^ requirements. Without addition of exogenous NAD^+^, the reaction produced 1.3 g L^−1^ UDP-GlcA, representing 100% and 46% conversion of UDP-Glc and UTP respectively. Finally, an anion exchange chromatography purification method was developed. UDP-GlcA was successfully obtained from the cascade system. The yield of UDP-GlcA during purification was about 92.0%.

**Conclusions:**

This work built a de novo hyperthermophilic biosynthetic cascade into *E. coli* host cells, with the cells able to meet NAD^+^ cofactor requirements and act as microbial factories for UDP-GlcA synthesis, which opens a door to large-scale production of cheaper UDP-GlcA.
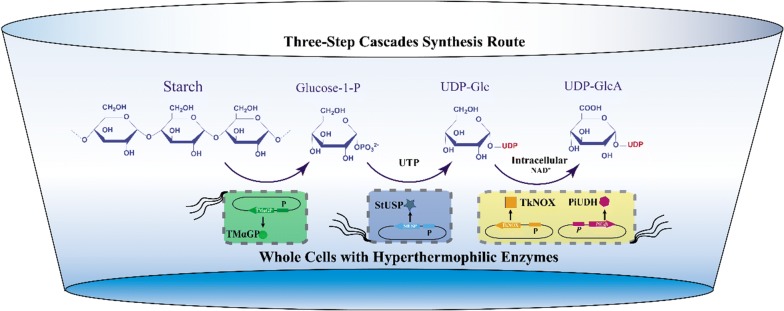

**Electronic supplementary material:**

The online version of this article (10.1186/s12934-019-1168-z) contains supplementary material, which is available to authorized users.

## Introduction

Glucuronic acid (GlcA) is one of the common building blocks of polysaccharides, proteoglycans and glycoglycerolipids [1]. GlcA can be transferred by glucuronosyltransferase (EC 2.4.1.17) to substrate molecules that contain oxygen, nitrogen, sulfur or carboxyl functional groups [2]. The uridine 5′-diphosphate (UDP) activated form, UDP-GlcA, is the monosaccharide donor for these glucuronidation reactions [2]. UDP-GlcA is one of nine nucleotide donor sugars used by glycosyltransferases in mammals. It is biosynthesized via two pathways. One involves UDP-sugar pyrophosphorylase (EC:2.7.7.-, USP), catalyzed directly from the respective GlcA-1-phosphates (GlcA1P). The other pathway, common in bacteria and animals, is synthesis of UDP-GlcA from UDP-glucose (UDP-Glc) by UDP-glucose 6-dehydrogenase (EC 1.1.1.22, UDH) using nicotinamide adenine dinucleotide (NAD^+^) as a cofactor [3, 4].

Understanding and application of enzymatic glycan synthesis has leapt forward in recent years [5–8]. Even an automated system for oligosaccharides synthesis is going to become possible [9, 10]. Meanwhile, a number of glycosyltransferases using UDP-GlcA as sugar donor have been identified and prepared [11–15], which provides a guide to an efficient approach to prepare glycans containing GlcA residues [16–19]. As the types and preparation scale of these oligosaccharides increase, the demand for UDP-GlcA is also increasing. However, natural nucleotide sugars are generally only present in plant and mammalian cells at pmol or nmol concentrations [20, 21], which is too low for them to be obtained directly. Two enzymatic approaches for UDP-GlcA synthesis from monosaccharide (Glc or GlcA) via in vitro multiple-enzyme pathways have been developed by Xi Chen’s group [22, 23]. In 2013, UDP-GlcA was prepared through a dehydrogenation step [22]. The newer system, reported in 2015, avoided the use of the expensive NAD^+^ cofactor via de novo synthesis from GlcA by *Arabidopsis thaliana* glucuronokinase and USP [23]. These systems have been successfully employed in oligosaccharide synthesis. However, the required purification steps limit the synthetic effectiveness and scale of the process, and push up the cost. The use of enzyme-cascades in whole cells is an effective strategy to produce UDP-GlcA in a more cost-effective way, because of the benefits of decreasing enzyme purification costs and potentially providing biochemical cofactors via endogenous pathways [24].

**Fig. 1:**
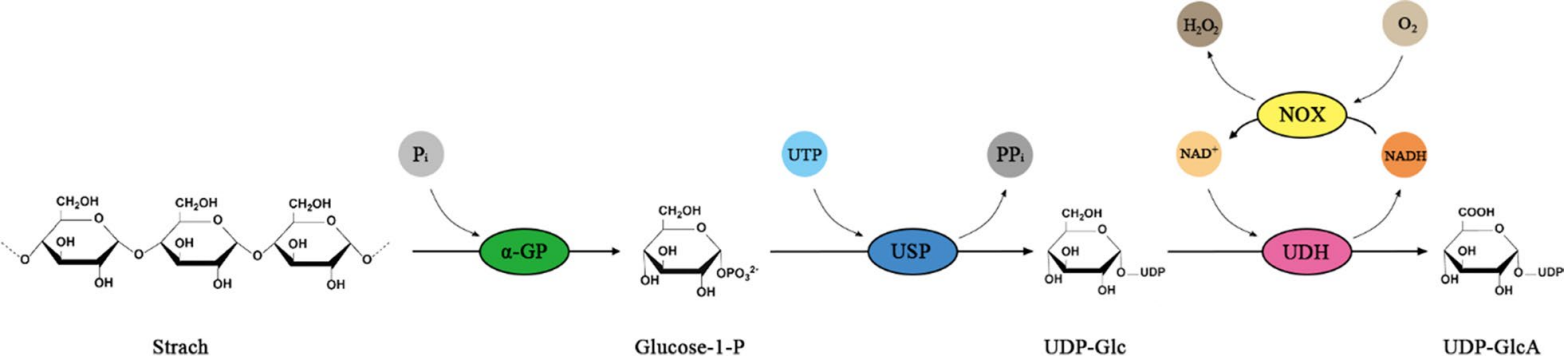
Scheme for synthesis of UDP-GlcA from starch by cascade biocatalysis. *α-GP* a-glucan phosphorylase, *USP* UDP-sugar pyrophosphorylase, *UDH* uronate dehydrogenase, *NOX* NADH oxidase

Therefore, we propose an added-cofactor-free whole-cell catalytic approach that converts starch to UDP-GlcA (Fig. 1). This pathway contains two steps: (1) phosphorylation of starch to glucose 1-phosphate (G1P) catalyzed by α-glucan phosphorylase; (2) conversion of G1P to UDP-Glc catalyzed by USP; and (3) dehydrogenation of UDP-Glc to UDP-GlcA catalyzed by UDH. Finally, an efficient three-step cascade route was developed using whole cells expressing hyperthermophilic enzymes, and valuable UDP-GlcA was produced in this cost-effective way from starch.

## Results and discussion

### Hyperthermophilic enzymes were soluble overexpressed well in whole-cell biocatalysts

To develop an efficient three-step cascade route and produce valuable UDP-GlcA from starch in a cost-effective way, thermophilic enzymes from hyperthermophilic microorganisms were employed. Since the activities of endogenous enzymes of *Escherichia coli* were sharply decreased at high temperature (> 70 °C) [25], and which avoids unwanted G1P, UTP or UDP-sugar consumption catalyzed by these endogenous enzymes. In addition, the molecular weight of the initial substrate (starch) is high. The permeability of the membrane of recombinant cells could be increased at high temperature, resulting in an increased probability of this substrates interacting with the catalytic enzymes.

The efficiency of formation of UDP-Glc is critical to the performance of our proposed cascade synthesis approach. One thermophilic USP, named StUSP, from *Sulfolobus tokodaii*, has been identified [26], and was used as the catalyst in this study. USPs, including StUSP, convert monosaccharides to UDP-sugars in a reversible reaction [26]. Therefore, to quantify the catalytic efficiency at high temperate, the kinetic parameters of StUSP and its conversion efficiency of G1P to UDP-Glc were determined (Additional file 1: Table S1 and Figure S1), demonstrating that this enzyme shows high specific activity and an acceptable level of to G1P at 80 °C.

**Fig. 2:**
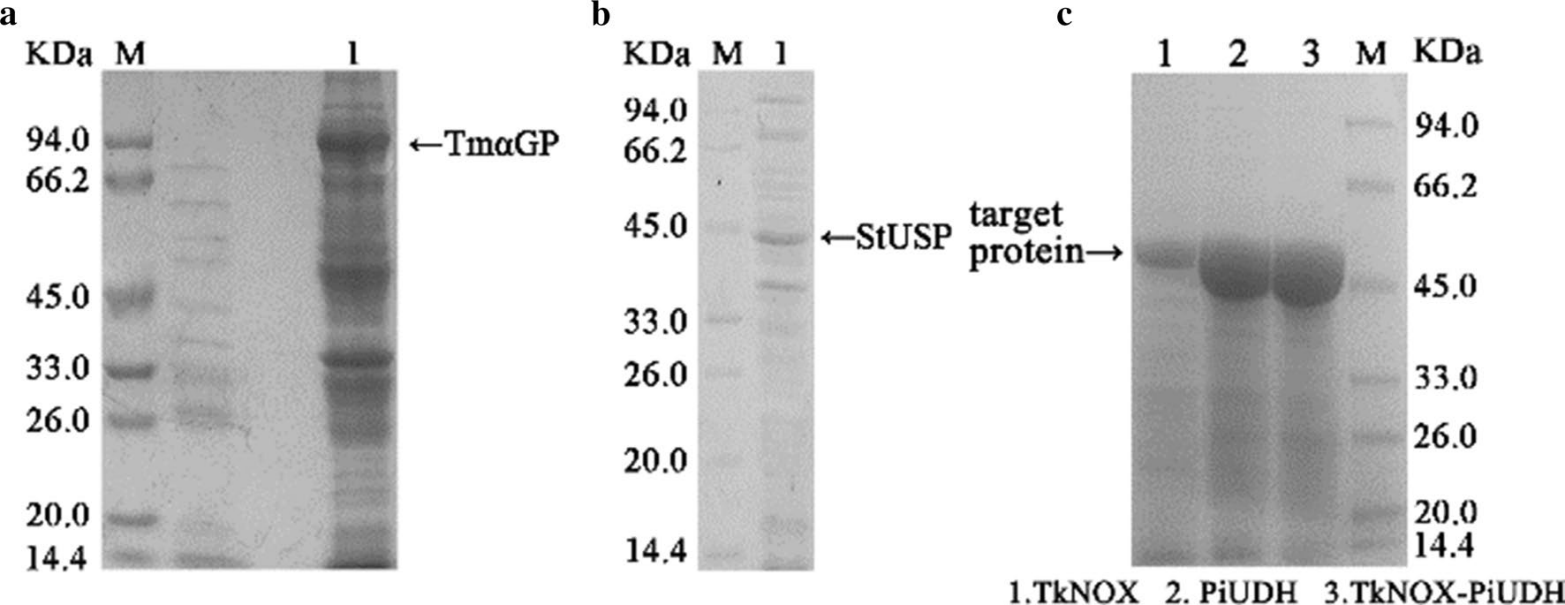
SDS-PAGE analysis of recombinant thermostable enzyme expression in whole cell catalysts. Harvested cells were heated at 80 °C for 20 min and then centrifuged at 12,000×*g*, then the supernatants were detected by 10% SDS-PAGE

Another three thermophilic enzymes—α-glucan phosphorylase (TmαGP) from *Thermotoga maritima* [27], PiUDH from *Pyrobaculum islandicum* [28], and TkNOX from *Thermococcus kodakarensis* [29]—were selected for use in this study because of their high thermostability and specific activities. Expression of these three enzymes, plus StUSP, encoded by codon-optimized genes, was induced with isopropyl-β-d-1-thiogalactopyranoside and SDS-PAGE analysis confirmed that all the enzymes were soluble and overexpressed well in *E. coli* (Fig. 2). It is worth noting that folding of thermophilic enzymes can be impaired in a mesophilic cytoplasmic environment by a number of factors, including temperature, but also by more subtle effects, such as translation kinetics of native versus optimized genes [30, 31].

### Whole-cell synthesis of UDP-Glc by a hybrid cascade system

**Fig. 3:**
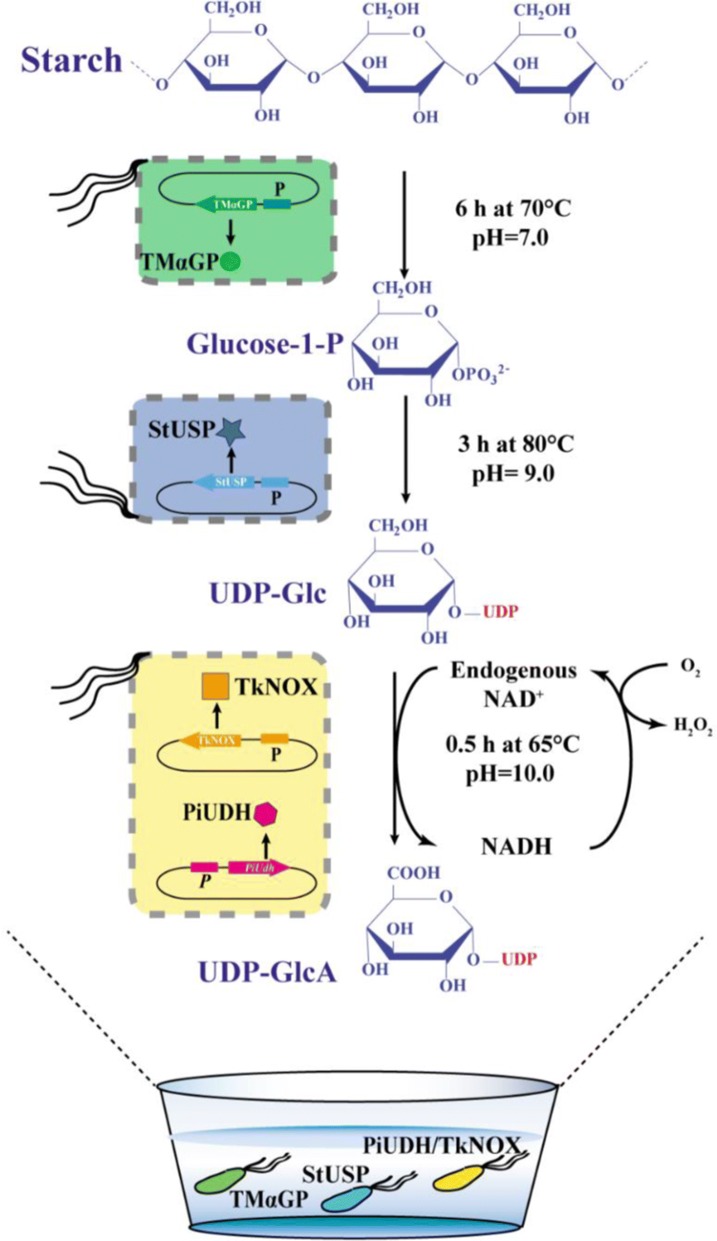
Scheme for the three-step cascade route using whole cells expressing hyperthermophilic enzymes to afford UDP-GlcA from starch

As illustrated in Fig. 3, a hybrid cascade system was used, comprised of two separate whole cell catalysts containing TmαGP and StUSP, respectively. First, a series of assays was carried out to determine the effects of temperature, pH and reaction time on the enzyme activity and stability of the whole-cell TmαGP-containing system when a preheated 5% (w/v) solution of soluble starch was used as substrate (Additional file 1: Figure S2). Gratifyingly, in the optimal conditions, the concentration of G1P in the reaction mixture reached approximately 40 mM (Table 1, entry 1); and 1 g G1P could be produced by the cells isolated from a 0.81 L fermentation. The optimal temperature was around 70 °C, close to the desired temperature (80 °C) of the follow-up step using the whole-cell StUSP system. These results thereby demonstrated the utility and potential of whole cells containing TmαGP in a cascade system for the production of UDP-Glc.

**Table 1:** Synthesis of UDP-GlcA from starch through a cascade using whole cells expressing hyperthermophilic enzymes

Entry	Pro .	Sub.	Sub. conc. (mM)	NAD^+^ (mM)	UTP (mM)	Whole-cells (DCW, g/L)					Time (h)	Conv. of sub. (%)	Conc. of product (mM)
1^a^	G1P	Starch	5% (w/v)	–	–	TmαGP (9.2)	–	–	–	–	6	22	40
2^b^	UDP-Glc	G1P	10	–	10	TmαGP (2.3)	StUSP (13.6)	–	–	–	3	46	4.6
3^c^	UDP-GlcA	UDPG	2.3	–	–	TmαGP (1.1)	StUSP (6.8)	PiUDH (14.6)	–	–	0.5	52	1.2
4^c^	UDP-GlcA	UDPG	2.3	0.1	–	TmαGP (1.1)	StUSP (6.8)	PiUDH (14.6)	–	–	0.5	100	2.3
5^c^	UDP-GlcA	UDPG	2.3	–	–	TmαGP (1.1)	StUSP (6.8)	PiUDH (14.6)	TkNOX (8.8)	–	0.5	100	2.3
6^c^	UDP-GlcA	UDPG	2.3	–	–	TmαGP (1.1)	StUSP (6.8)	–	–	PiUDH-TkNOX (17.8)	0.5	100	2.3

Data are the average of triplicate measurements

^a^Entry 1 was incubated in a reaction mixture (pH 7.0) at 70 °C for 6 h

^b^Entry 2 was incubated in a reaction mixture (pH 9.0) at 80 °C for 3 h

^c^Entries 3, 4, 5 and 6 were incubated in a reaction mixture (pH 10.0) at 65 °C for 0.5 h

**Fig. 4:**
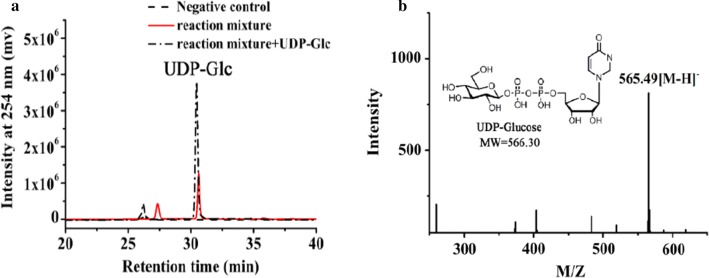
Analysis of UDP-Glc formation. **a** The supernatant of reaction catalyzed by the hybrid cascade system comprised of separate whole cell catalysts expressing TmαGP and StUSP, in the optimized conditions described above, was assayed by PAMN-HPLC. **b** ESI–MS spectrum of product UDP-Glc. The supernatant of reaction catalyzed by the hybrid cascade system comprised of separate whole cell catalysts expressing TmαGP and StUSP, in the optimized conditions described above, was assayed by MS after desalting using a P2 column

Next, whole cells containing StUSP were introduced into the above mixture to produce UDP-Glc. The optimal conditions for this were determined (Additional file 1: Figures S3–S7): the optimal temperature was around 80 °C, which is consistent with data for purified StUSP; the optimal pH was around 9.0, obviously greater than that (~ 7.5) for the purified enzyme. It appears that an alkaline environment makes the cell membranes more permeable to substrate, and benefits overall UDP-Glc synthesis in our system. The product of the TmαGP-catalyzed step should be diluted fourfold with phosphate buffer and the pH adjusted to 9.0, making the concentration of G1P approximately 10 mM. The price of UTP is higher than the cost of preparation of G1P and the whole-cell StUSP catalyst, thus, more efficient use of UTP will lead to better economic performance of our proposed cascade synthesis approach. However, the conversion of UTP is difficult to exceed 50%, even if the amount of whole cells and the ratio of UTP to G1P was increased in the reaction system (Additional file 1: Figure S7). This conclusion is consistent with data for in vitro reaction catalyzed by purified StUSP, demonstrating that the reversible nature of the USP reaction is likely one of the limiting factors affecting the performance of UDP-sugar synthesis, and this needs to be addressed in the future. USP mutants created by protein engineering could be a strategy to address this issue. The product concentration in our system was determined by polyamine-based anion exchange–high-performance liquid chromatography (PAMN-HPLC) (Fig. 4a) [32], and the identity of UDP-Glc was confirmed by electrospray ionization-mass spectrometry (ESI–MS) as described previously [33] (Fig. 4b). We obtained 4.6 mM UDP-Glc in the reaction mixture, with 46% conversion of UTP (Table 1, entry 2).

### Synthesis of UDP-GlcA using an added-cofactor-free whole-cell catalytic approach

**Fig. 5:**
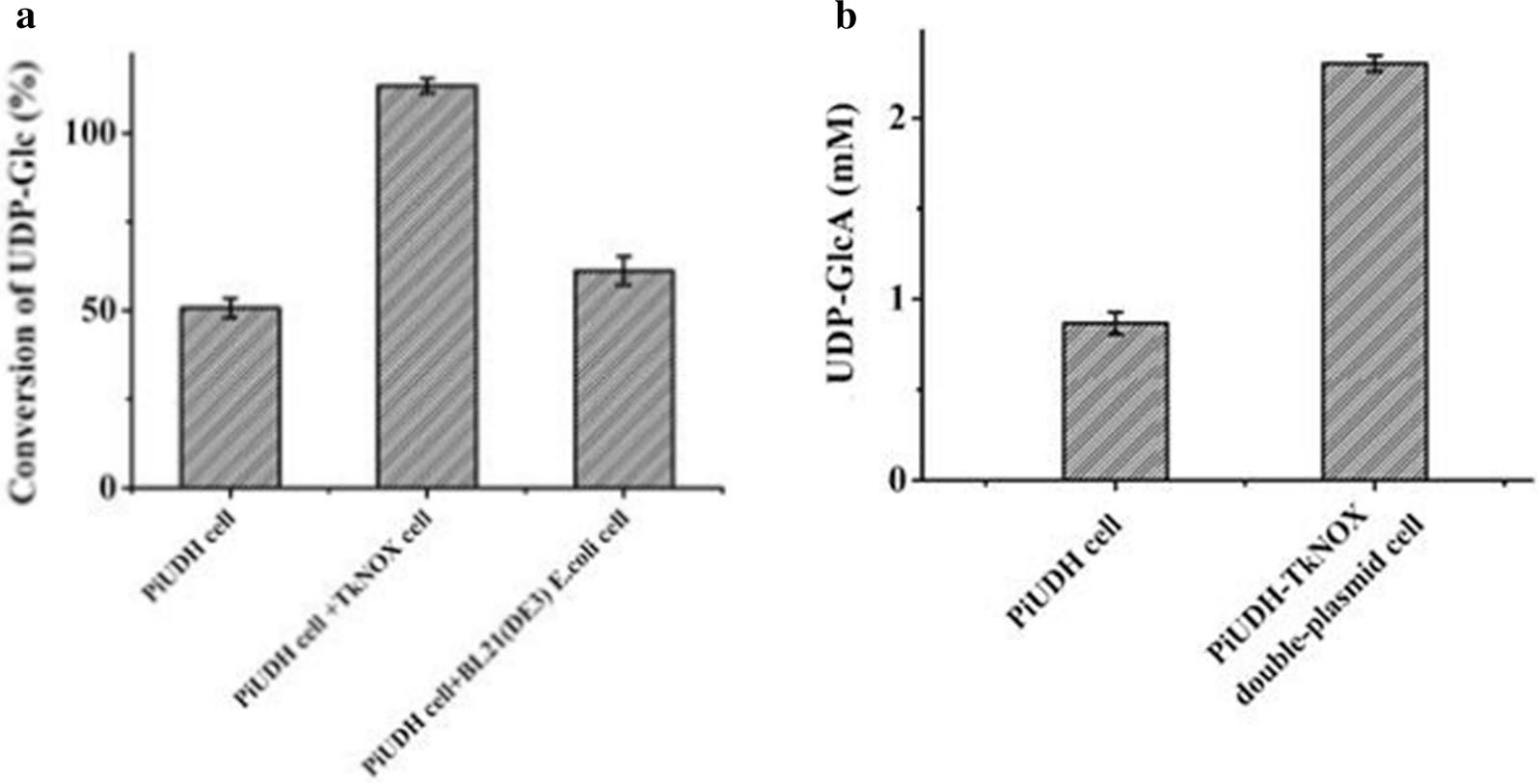
Effect of NAD^+^ regeneration system on UDP-GlcA formation. **a** BL21-PiUDH, BL21-TkNOX and plasmid-free *E. coli* BL21 (DE3) cells were fermented and used as catalysts. Initially, BL21-PiUDH cells were incubated in a mixture containing 2.3 mM UDP-Glc, 0 mM NAD^+^ and 200 mM sodium phosphate (pH10). Then, groups containing the same components, and an additional BL21-TkNOX or BL21 cells respectively, were introduced into the reactions. These mixtures were incubated for 0.5 h and supernatants were detected by HPLC. **b** BL21-PiUDH and BL21-PiUDH-TkNOX cells were fermented and used as catalysts. BL21-PiUDH or BL21-PiUDH-TkNOX cells were incubated with 2.3 mM UDP-Glc, without extra supplementation, at 65 °C for 0.5 h. These mixtures were incubated for 0.5 h and supernatants were detected by HPLC. And bars indicate the range of assay results from three different batches

**Fig. 6:**
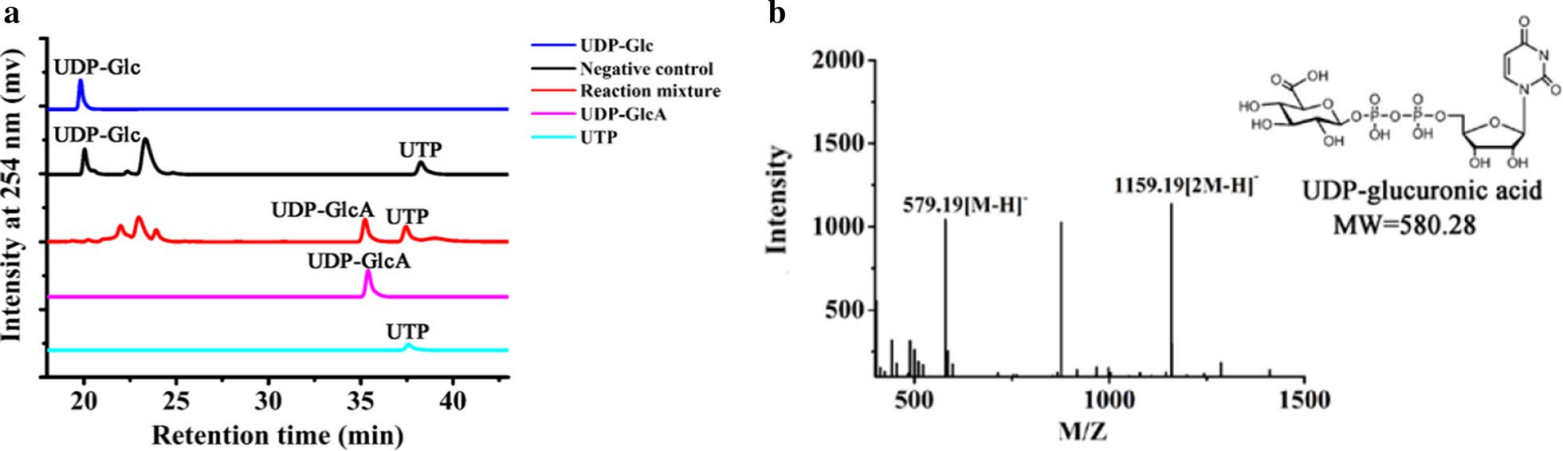
Analysis of UDP-GlcA formation. **a** The supernatant of the three-step cascade reaction was assayed by PAMN-HPLC. **b** ESI–MS of product UDP-GlcA. The supernatant of the three-step cascade reaction was assayed by MS after desalting using a P2 column

Whole cells containing both the thermophilic UDP-Glc dehydrogenase (PiUDH) and an NAD^+^ regeneration system (TkNOX) were introduced into the reaction mixture of step 2, resulting in an added-cofactor-free whole-cell catalytic approach. NAD^+^, an oxidizing cofactor of UDP-Glc dehydrogenase, accepts electrons from UDP-Glc and becomes reduced to NADH. NAD^+^ is a costly compound if added to synthesis systems. A series of assays was carried out to determine the effects of NAD^+^ concentration, as well as other reaction conditions, on the UDP-GlcA yield (Additional file 1: Figure S8–S11). The reaction mixture from the TmαGP and StUSP cascade system was used as substrate. There was nearly 50% conversion of UDP-Glc to UDP-GlcA in reaction without addition of exogenous NAD^+^ (Table 1, entry 3 and Additional file 1: Figure S11), which indicated that intracellular NAD^+^ played a role in the dehydrogenase reaction. The synthetic efficiency showed a positive correlation with the concentration of NAD^+^ in the thermophilic system, and UDP-Glc could be fully converted into UDP-GlcA when enough NAD^+^ was added (Table 1, entry 4 and Additional file 1: Figure S11). Therefore, a thermophilic NAD^+^ regeneration system was employed to make our approach added-cofactor-free [34]. Firstly, whole cells overexpressing TkNOX were constructed (Fig. 1d), and its NAD^+^ regeneration role was confirmed. The UDP-Glc conversion reached nearly 100% in the hybrid cascade system containing two separate types of whole cells expressing TkNOX and PiUDH respectively (Table 1, entry 5 and Fig. 5a). The number of TkNOX-containing cells in this system was approximately half that of the PiUDH-containing cells. Both genes were expressed under the control of the same promotor (the T7 promoter), and in the same plasmid system (the pET system). Therefore, next, a low-copy-number plasmid with the T7 promoter, pACYCDuet-1, was used to express TkNOX in PiUDH-expressing cells, resulting in a strain coexpressing these two enzymes (Figs. 1e and 5b). Then, the optimal conditions for this whole cell catalytic system were determined (Additional file 1: Figure S12), and the identity of the synthetic products was confirmed by (PAMN)-HPLC and ESI–MS (Fig. 6). We obtained 1.3 gL^−1^ UDP-GlcA without addition of exogenous NAD^+^, with 100% conversion of UDP-Glc (Table 1, entry 6).

### Purification of UDP-GlcA from the cascade system

**Fig. 7:**
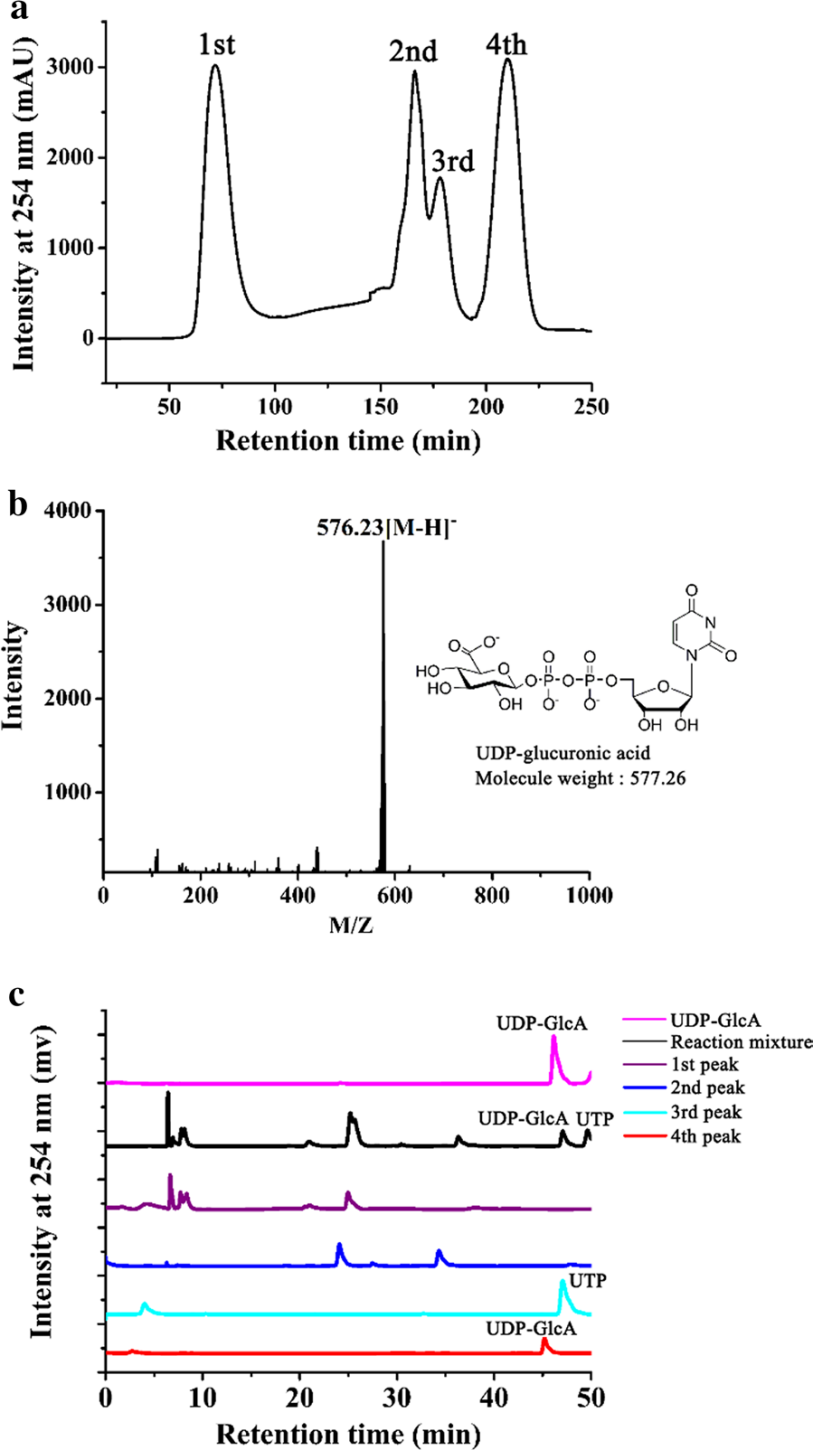
Purification of UDP-GlcA. **a** Anion exchange chromatography profile of the products of the three-step cascade. **b** ESI–MS spectrum of the fourth peak eluted from the anion exchange column. **c** PAMN-HPLC of fractions eluted from the anion exchange column, as well as the reaction mixture and a UDP-GlcA standard

We developed a purification method for UDP-GlcA from the cascade system, based on anion exchange chromatography. Four peaks were seen as the purification proceeded (Fig. 7a). The fractions were desalted and subjected to HPLC and ESI–MS analysis. A compound with a molecular mass of 577.23 Da was identified in fractions of the fourth peak eluted from the anion exchange column, which is very close to the calculated molecular mass of UDP-GlcA, 577.26 Da (Fig. 7b). In high-resolution HPLC, this product showed a single symmetric peak and eluted at 42 min (Fig. 7c). These results suggested that UDP-GlcA was successfully obtained. The yield of UDP-GlcA during purification was about 92.0% (i.e., loss of < 10%). This method can also be applied to purify UDP-Glc from the products of step 1 (unpublished data).

## Conclusions

This work built a de novo hyperthermophilic biosynthetic cascade into *E. coli* host cells, with the cells able to meet NAD^+^ cofactor requirements and act as microbial factories for UDP-GlcA synthesis. From simple starting materials, 1 g of valuable UDP-GlcA could be obtained by using approximately 1.8 g UTP, suggesting this three-step cascade route opens a door to large-scale production of UDP-GlcA at lower cost than previous methods.

## Methods

### General procedures for preparation of whole-cell biocatalysts

The plasmids involved in the construction of recombinant *Escherichia coli* cells and the strains are listed in Table 2.

**Table 2:** Plasmids and strains used in this study

Strain or plasmid	Characteristics	Source
*E. coli* strains		
DH5α		Thermo Fisher
BL21 (DE3)		Thermo Fisher
BL21-TmαGP	pET20b-TmαGP	This work
BL21-StUSP	pET21b-StUSP	This work
BL21-PiUDH	pET15b-PiUDH	This work
BL21-TkNOX	pET21b-TkNOX	This work
BL21-PiUDH-TkNOX	pET15b-PiUDH and pACYCDuet1-TkNOX	This work
Plasmids		
pET20b(+)	Expression vector, Ap^R^	Novagen
pET21b(+)	Expression vector, Ap^R^	Novagen
pET15b	Expression vector, Ap^R^	Novagen
pACYCDuet-1	Expression vector, Cm^R^	Novagen
pET20b-TmαGP	Expression vector of TmαGP	This work
pET21b-StUSP	Expression vector of ST0452	This work
pET15b-PiUDH	Expression vector of PiUDH	This work
pET20b-TkNOX	Expression vector of TkNOX	This work
pACYC Duet1-TkNOX	Expression vector of TkNOX	This work

The *TmαGP* gene (GenBank accession number NC_000853.1), *StUSP* gene (GenBank accession number NC_003106.2), *PiUDH* gene (GenBank accession number NC_008701.1), and *TkNOX* gene (GenBank accession number NC_006624.1) with a 5ʹ-His tag fragments were synthesized with codon optimization for gene expression in *E. coli* by GenScript Inc. (Nanjing, China), respectively. Then these enzymes were recombinant expression in *E. coli* BL21 (DE3) cells as soluble N-His6-tagged fusion proteins and purified by appropriate affinity chromatography, as described in Additional file 1.

The *TkNOX* gene DNA fragment was also inserted into vector pACYC Duet-1 (Novagen). This plasmid was named pACYC Duet1-tknox, and was introduced into *E. coli* BL21-PiUDH, producing recombinant strain BL21-PiUDH-TkNOX.

These recombinant strains were grown at 37 °C in high density fermentation medium with 100 μg/mL ampicillin, and only PiUDH-TkNOX double-plasmid recombinant *E. coli* use two antibiotics, 100 μg/mL ampicillin and 34 μg/mL chloromycetin. In fermentation process, pH should be controlled to 7.0, stirring speed was 500 rpm, and dissolved oxygen content should be controlled to 25% of maximum value of dissolved oxygen content. Cells were then cultured until optical density at 600 nm (OD_600_) increased to 15–20. 1 mM isopropyl-b-d-thiogalactopyranoside (IPTG) was used to induce *E. coli* at 22 °C for more than 20 h.

### Optimization of whole-cell synthesis of G1P and final optimized conditions

Reaction was in a 500 μL mixture including 5% (w/v) soluble starch, 0.7 M potassium phosphate buffer (pH7.0) and about 3.3 mg (DCW) cells expressing TmαGP. Reaction was terminated by chilling on ice for 5 min, and then the mixture was centrifuged at 12,000×*g* for 20 min at 4 °C. Then, the supernatant was precipitated with 0.7 M Ca^2+^ (CaCl_2_) to remove phosphate and then diluted to twice the original volume before detection by thin-layer chromatography (TLC). TLC analysis was performed on TLC Silica gel 60 F_254_ (Merck KGA, Germany), developed by *n*-butanol:acetic acid:ddH_2_O (2:1:1, v/v), and subsequently stained with anisaldehyde. G1P shows as a dark spot after being stained. As shown in Additional file 1: Figure S2, incubating at 70 °C for 6 h at pH 7.0 was determined to be optimal for TmαGP in whole-cell catalysis.

### Catalytic analysis of purified StUSP

Strain *BL21*-*StUSP* was cultured, and treated with 0.2 mM IPTG for 18–20 h at 22 °C to induce expression of StUSP. Then, N-terminally His-tagged recombinant *StUSP* was purified using Ni^2+^-affinity resin, and incubated with substrates at 80 or 37 °C. The forward reaction of StUSP was performed in a 200-μL mixture including 2 mM G1P, 20 mM MgCl_2_, 50 mM Tris–HCl (pH 7.5), 3 mM UTP, and 1 μg purified enzyme. The reverse reaction was performed in a 200-μL mixture including 0.5 mM UDP-Glc, 2 mM MgCl_2_, 50 mM Tris–HCl (pH 7.5), 1 mM pyrophosphate, and 1 μg of purified enzyme. After the reactions reached equilibrium, the products were resolved via (PAMN)-HPLC. The absorbance at 254 nm, which corresponds to the UDP group, was used to monitor the products.

To measure the *K*_*m*_ and *V*_*max*_ values of the forward reaction, reactions were carried out at G1P concentrations varying from 0.2 to 3 mM in a buffer described above. Six time-response samples were collected for donor concentrations and obtained by (PAMN)-HPLC, then fitted to the Michaelis–Menten equation.

### Optimization of whole-cell synthesis of UDP-Glc

To determine the effects of temperature on UDP-Glc formation, BL21-TmαGP cells (12.5 mg DCW per milliliter) were incubated in a mixture consisting of 2 mM G1P, 3 mM UTP, 20 mM Mg^2+^, and 50 mM sodium phosphate (pH9.0) at 60, 70, 75, 80, 85, 90, and 95 °C, respectively. The reactions were terminated by chilling on ice for 5 min, centrifuged at 12,000 rpm for 20 min at 4 °C, and the supernatants were detected by HPLC. Reactions were performed to determine effect of the pH (in the range 2.5–12), reaction time (0, 5, 10, 30, 40, 60, 80, 120, 180 and 240 min), and the dry cell weight added (0, 0.09, 0.23, 0.46, 0.91, 1.37 and 1.82 mg) similar to that described for the optimized temperature assay. Each group of the reaction was performed using three paralleled assays.

### Optimization of whole-cell synthesis of UDP-GlcA

The effects of temperature of whole-cell synthesis of UDP-GlcA were investigated using UDP-Glc as substrate. BL21-PiUDH cells (17.5 mg DCW per milliliter) were incubated in a mixture consisting of 2.3 mM UDP-Glc, 1.5 mM NAD^+^ and 200 mM sodium phosphate (pH10) at 50, 60, 65, 70, 75, 80, and 85 °C, respectively. Similarly, multiple reactions with various pH values, reaction time, dry cell weight added, and cofactor concentration were carried out simultaneously with all of the other components fixed. Each group of the reaction was performed using three paralleled assays.

### NAD^+^/NADH regeneration system

BL21-PiUDH, BL21-TkNOX and plasmid-free *E. coli* BL21 (DE3) cells were fermented and used as catalysts. Initially, BL21-PiUDH cells (14.5 mg DCW per milliliter) were incubated in a mixture containing 2.3 mM UDP-Glc, 0 mM NAD^+^ and 200 mM sodium phosphate (pH10). Then, groups containing the same components, and an additional BL21-TkNOX or BL21 cells (7.25 mg DCW per milliliter) respectively, were introduced into the reactions. In addition, BL21-PiUDH and BL21-PiUDH-TkNOX cells were fermented and used as catalysts. BL21-PiUDH or BL21-PiUDH-TkNOX cells (17.5 DCW per milliliter) were incubated with 2.3 mM UDP-Glc, without extra supplementation, at 65 °C for 0.5 h. The supernatants were detected by HPLC.

### Effect of dry cell weight addition of BL21-PiUDH-TkNOX cells on UDP-GlcA formation

UDP-Glc (2.3 mM) was respectively incubated with 0, 0.08, 0.16, 0.40, 0.79, 1.19, 1.58, 2.37, 3.17, 4.75, 6.33 and 7.12 mg BL21-PiUDH-TkNOX cells (DCW), without extra supplementation in 400 μL mixture, at 65 °C for 0.5 h. The supernatants were detected by HPLC. All experiments were repeated three times.

### Purification of UDP-GlcA

The purification process was performed using an Äkta Avant 150 (GE Healthcare Bio-Sciences AB, Uppsala, Sweden) with a Q-Sepharose Fast-Flow column, and the pH of the reaction mixture and the buffers was adjusted to 3.7. The column was first eluted with a linear gradient of NaCl from 0 to 0.25 M over one column volume, and then with 0.25 M NaCl for one column volume, and finally with one column volume of 0.3 M NaCl. The flow rate throughout the process was 2.5 mL/min.

### High-resolution HPLC analysis

PAMN-HPLC analysis was performed using a Shimadzu HPLC instrument (Tokyo, Japan) equipped with a YMC-Pack Polyamine II column (250 × 4.6 mm; Shimogyo-ku, Kyoto, Japan) [14]. The column was eluted at a flow rate of 0.5 mL/min, with a linear gradient of KH_2_PO_4_ from 0 to 0.6 M over 40 min for UDP-Glc detection, and a linear gradient of KH_2_PO_4_ from 0.2 to 0.6 M over 40 min for UDP-GlcA detection. The absorbance at 254 nm, from the UDP group, was used to monitor the products.

### Mass spectrometry

MS analysis was performed on a Thermo LCQ Deca mass spectrometer. The experiments were performed using negative ionization mode with a spray voltage of 3 kV and a capillary temperature of 275 °C [33]. MS data were acquired and processed using Xcalibur 1.3 software.

## Additional file

**Additional file 1: Table S1.** Effect of temperature on kinetics of purified StUSP. **Figure S1.** Kinetic parameters of, and substrate conversion by, purified StUSP. Determination of kinetic parameters of StUSP at 37 °C (A) and 80 °C (B). G1P was used as substrate to determine the kinetic parameters of purified StUSP. The amounts of product UDP-Glc were determined by PAMN-HPLC. (C) Substrate conversion in activity assays of purified StUSP at 37 and 80 °C. All data represent the average of three independent determinations. **Figure S2.** Optimization of the reaction catalyzed by whole cells expressing TmαGP. A, Temperature optimization. Lanes 1 to 5, reactions were carried out at 50, 60, 70, 80, and 90 °C, respectively. Lane 6, G1P standard (20 mM). B, TLC assays of whole-cell TmαGP catalysis at various pHs. Lanes 1 to 10, reactions were carried out in a mixture adjusted to pH 3, 4, 5, 6 ,7, 8, 9, 10, 11, and 12, respectively; Lane 6 and 11, G1P standard (20 mM). C, Effect of reaction time on G1P formation. Lanes 1 to 11, reactions were performed for 0, 5, 10, 20, 30, 60, 120, 180, 360, 660, and 960 min, respectively; Lane 12, G1P (20 mM). **Figure S3.** Analysis of StUSP catalysis in whole-cells by temperature. BL21-TmαGP cells (12.5 mg DCW per milliliter) were incubated in a mixture consisting of 2 mM G1P, 3 mM UTP, 20 mM Mg^2+^, and 50 mM sodium phosphate (pH9.0) at 60, 70, 75, 80, 85, 90, and 95 °C, respectively. The reactions were terminated by chilling on ice for 5 min, centrifuged at 12000 rpm for 20 min at 4 °C, and the supernatants were detected by HPLC. Bars indicate the range of assay results from three different batches. **Figure S4.** pH profile of StUSP catalysis in whole cells. Reactions were performed in a mixture consisting of 2 mM G1P, 3 mM UTP, 20 mM Mg^2+^, 50 mM sodium phosphate, and about BL21-TmαGP cells (12.5 mg DCW per milliliter), at various pH values in the range 2.5–12. Bars indicate the range of assay results from three different batches. **Figure S5.** Effect of reaction time on UDP-Glc formation. Reactions were performed in a mixture consisting of 2 mM G1P, 3 mM UTP, 20 mM Mg^2+^, 50 mM sodium phosphate (pH9.0), and about BL21-TmαGP cells (12.5 mg DCW per milliliter), for 0, 5, 10, 30, 40, 60, 80, 120, 180 and 240 min, respectively. Bars indicate the range of assay results from three different batches. **Figure S6.** Activity assays of whole cells expressing StUSP by quantity of cells added. Glc-1-P (2 mM) was respectively incubated with 0, 0.09, 0.23, 0.46, 0.91, 1.37 and 1.82 mg (DCW) BL21-TmαGP cells in a 320-μL mixture consisting of 3 mM UTP, 20 mM Mg^2+^and 50 mM sodium phosphate (pH9.0). All experiments were repeated three times. About 4.28 g DCW of StUSP-expressing cells/L reaction mixture was determined to be the most suitable value for UDP-Glc formation. **Figure S7.** Optimization of usage of UTP in UDP-Glc formation by BL21-TmαGP cells. BL21-TmαGP cells (4.3 mg DCW per milliliter)) were incubated with the substrate UTP, the concentration of which was varied from 0 to 6 mM, in a mixture containing 2 mM G1P, 20 mM Mg^2+^ and 50 mM sodium phosphate (pH9.0). Bars indicate the range of assay results from three different batches. **Figure S8.** Analysis of PiUDH catalysis in whole-cells by temperature. BL21-PiUDH cells (17.5 mg/mL DCW) were incubated in a mixture consisting of 2.3 mM UDP-Glc, 1.5 mM NAD^+^ and 200 mM sodium phosphate (pH10) at 50, 60, 65, 70, 75, 80, and 85 °C, respectively. The reactions were terminated by chilling on ice for 5 min, and then centrifuged at 12000×*g* for 20 min at 4 °C, and the supernatants were detected by HPLC. Bars indicate the range of assay results from three different batches. **Figure S9.** pH profile of production of UDP-GlcA by whole cells expressing PiUDH. Reactions were performed in a mixture consisting of 2.3 mM UDP-Glc, 1.5 mM NAD^+^, 200 mM sodium phosphate, and 17.5 mg/mL (DCW) BL21-PiUDH cells at pH values in the range 2.5–13. Bars indicate the range of assay results from three different batches. **Figure S10.** Effect of reaction time on UDP-GlcA formation by whole cells expressing PiUDH. Reactions were performed in a mixture consisting of 2.3 mM UDP-Glc, 1.5 mM NAD^+^, 200 mM sodium phosphate (pH10), and BL21-PiUDH cells (17.5 mg DCW per milliliter), for 0, 10, 20, 30, 40, 60, 90, 120, 150, and 180 min, respectively. **Figure S11.** Optimization of usage of NAD^+^ in whole-cell catalysis by cells expressing PiUDH. BL21-PiUDH cells (17.5 mg DCW per milliliter) were incubated with the cofactor NAD^+^, the concentration of which was varied from 0 to 1.2 mM, in a mixture containing 2.3 mM UDP-Glc and 200 mM sodium phosphate (pH9.0) for 3 h. Bars indicate the range of assay results from three different batches. **Figure S12.** Activity assays of BL21-PiUDH-TkNOX cells by quantity of cells added. Bars indicate the range of assay results from three different batches.
