# Supplementary material for: Cascade synthesis of uridine-5′-diphosphate glucuronic acid by coupling multiple whole cells expressing hyperthermophilic enzymes
Source: Microb Cell Fact. 2019 Jul 1;18:118. doi: 10.1186/s12934-019-1168-z (PMC6604206; doi:10.1186/s12934-019-1168-z)
Supplement: Supplementary file 1 — Additional file 1: Table S1. Effect of temperature on kinetics of purified StUSP. Figure S1. Kinetic parameters of, and substrate conversion by, purified StUSP. Determination of kinetic parameters of StUSP at 37 °C (A) and 80 °C (B). G1P was used as substrate to determine the kinetic parameters of purified StUSP. The amounts of product UDP-Glc were determined by PAMN-HPLC. (C) Substrate conversion in activity assays of purified StUSP at 37 and 80 °C. All data represent the average of three independent determinations. Figure S2. Optimization of the reaction catalyzed by whole cells expressing TmαGP. A, Temperature optimization. Lanes 1 to 5, reactions were carried out at 50, 60, 70, 80, and 90 °C, respectively. Lane 6, G1P standard (20 mM). B, TLC assays of whole-cell TmαGP catalysis at various pHs. Lanes 1 to 10, reactions were carried out in a mixture adjusted to pH 3, 4, 5, 6 ,7, 8, 9, 10, 11, and 12, respectively; Lane 6 and 11, G1P standard (20 mM). C, Effect of reaction time on G1P formation. Lanes 1 to 11, reactions were performed for 0, 5, 10, 20, 30, 60, 120, 180, 360, 660, and 960 min, respectively; Lane 12, G1P (20 mM). Figure S3. Analysis of StUSP catalysis in whole-cells by temperature. BL21-TmαGP cells (12.5 mg DCW per milliliter) were incubated in a mixture consisting of 2 mM G1P, 3 mM UTP, 20 mM Mg2+, and 50 mM sodium phosphate (pH9.0) at 60, 70, 75, 80, 85, 90, and 95 °C, respectively. The reactions were terminated by chilling on ice for 5 min, centrifuged at 12000 rpm for 20 min at 4 °C, and the supernatants were detected by HPLC. Bars indicate the range of assay results from three different batches. Figure S4. pH profile of StUSP catalysis in whole cells. Reactions were performed in a mixture consisting of 2 mM G1P, 3 mM UTP, 20 mM Mg2+, 50 mM sodium phosphate, and about BL21-TmαGP cells (12.5 mg DCW per milliliter), at various pH values in the range 2.5–12. Bars indicate the range of assay results from three different batches. Figure S5. Effect of [file 12934_2019_1168_MOESM1_ESM.docx]

**Additional file 1**

**Cascade synthesis of uridine-5′-diphosphate glucuronic acid by coupling multiple whole cells expressing hyperthermophilic enzymes**

Dan-Hua Meng^1^, Ran-Ran Du^1^, Lu-Zhou Chen^1^, Meng-Ting Li^1^, Fei Liu^2^, Jin Hou^3^, Yi-Kang Shi^4^, Feng-Shan Wang^1,4^, Ju-Zheng Sheng*^1,4^

^1^ Key Laboratory of Chemical Biology of Natural Products (Ministry of Education), School of Pharmaceutical Sciences, Shandong University, Jinan 250012, China

^2^ Key Laboratory of Biopharmaceuticals, Shandong Academy of Pharmaceutical Sciences, Jinan 250101, China

^3^ State Key Laboratory of Microbiology, Shandong University, Jinan 250100, China

^4^ National Glycoengineering Research Center, Shandong University, Jinan 250012, China

**Corresponding author: Ju-Zheng Sheng, E-mail:* [*shengjuzheng@sdu.edu.cn*](mailto:shengjuzheng@sdu.edu.cn)

**Table S1 Effect of temperature on kinetics of purified StUSP**

| Reaction temperature | K*_m_* (mM) | V*_max_* (mmol/min/mg) | Conversion rate (%) |
| --- | --- | --- | --- |
| 37 °C | 1.079±0.139 ^a^ | 1.253±0.150 ^a^ | 6.99±1.03 ^c^ |
| 80 °C | 1.474±0.225 ^b^ | 14.456±0.227 ^b^ | 52.44±1.56 ^c^ |

*The percentage of Glc-1-P transformed to UDP-Glc was considered the reaction conversion rate.

All data represent the average of these three independent determinations.

^a^ see Supplemental Fig. S1A.

^b^ see Supplemental Fig. S1B.

^c^ see Supplemental Fig. S1C.


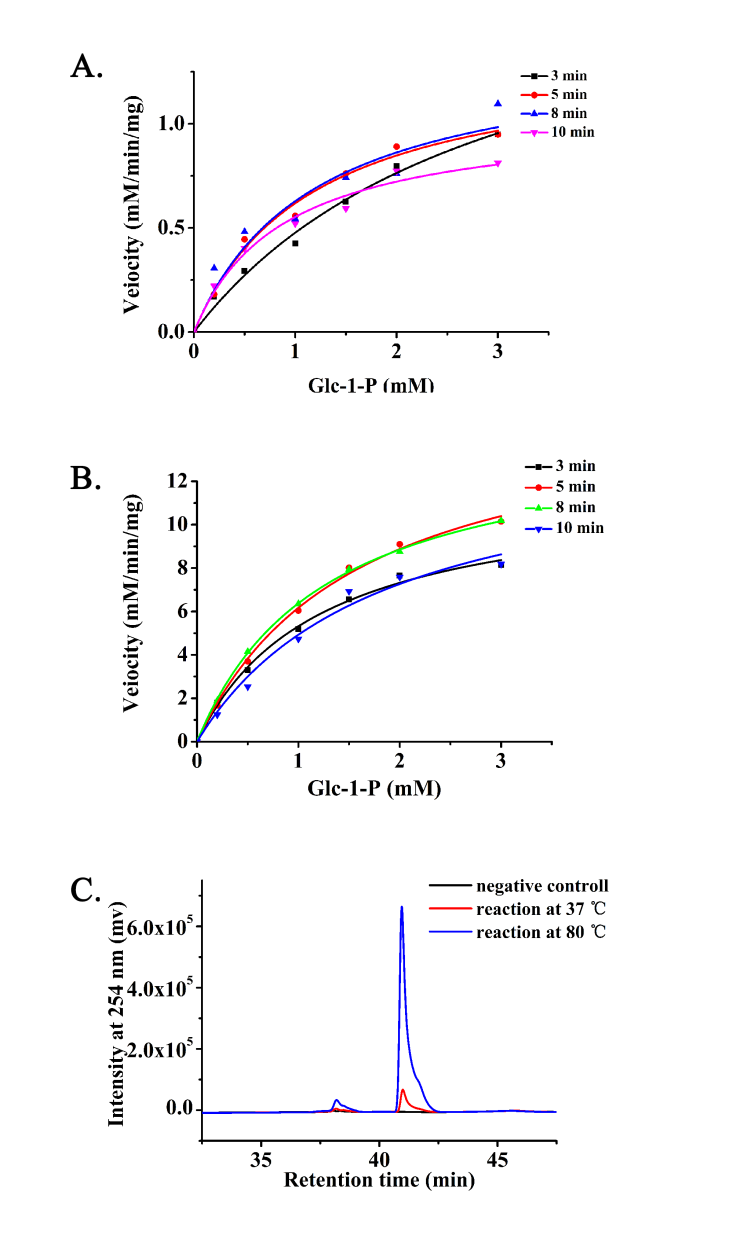


**Figure S1 Kinetic parameters of, and substrate conversion by, purified StUSP.** Determination of kinetic parameters of StUSP at 37 °C (A) and 80 °C (B). G1P was used as substrate to determine the kinetic parameters of purified StUSP. The amounts of product UDP-Glc were determined by PAMN-HPLC. (C) Substrate conversion in activity assays of purified StUSP at 37 and 80 °C. All data represent the average of three independent determinations.





Fig. S2 Optimization of the reaction catalyzed by whole cells expressing TmαGP. A, Temperature optimization. Lanes 1 to 5, reactions were carried out at 50, 60, 70, 80, and 90 °C, respectively. Lane 6, G1P standard (20 mM). B, TLC assays of whole-cell TmαGP catalysis at various pHs. Lanes 1 to 10, reactions were carried out in a mixture adjusted to pH 3, 4, 5, 6 ,7, 8, 9, 10, 11, and 12, respectively; Lane 6 and 11, G1P standard (20 mM). C, Effect of reaction time on G1P formation. Lanes 1 to 11, reactions were performed for 0, 5, 10, 20, 30, 60, 120, 180, 360, 660, and 960 min, respectively; Lane 12, G1P (20 mM).





Fig. S3 Analysis of StUSP catalysis in whole-cells by temperature. BL21-TmαGP cells (12.5 mg DCW per milliliter) were incubated in a mixture consisting of 2 mM G1P, 3 mM UTP, 20 mM Mg^2+^, and 50 mM sodium phosphate (pH9.0) at 60, 70, 75, 80, 85, 90, and 95 °C, respectively. The reactions were terminated by chilling on ice for 5 min, centrifuged at 12000 rpm for 20 min at 4 °C, and the supernatants were detected by HPLC. Bars indicate the range of assay results from three different batches.





Fig. S4 pH profile of StUSP catalysis in whole cells. Reactions were performed in a mixture consisting of 2 mM G1P, 3 mM UTP, 20 mM Mg^2+^, 50 mM sodium phosphate, and about BL21-TmαGP cells (12.5 mg DCW per milliliter), at various pH values in the range 2.5–12Bars indicate the range of assay results from three different batches.





Fig. S5 Effect of reaction time on UDP-Glc formation. Reactions were performed in a mixture consisting of 2 mM G1P, 3 mM UTP, 20 mM Mg^2+^, 50 mM sodium phosphate (pH9.0), and about BL21-TmαGP cells (12.5 mg DCW per milliliter), for 0, 5, 10, 30, 40, 60, 80, 120, 180 and 240 min, respectively. Bars indicate the range of assay results from three different batches.





Fig. S6 Activity assays of whole cells expressing StUSP by quantity of cells added. Glc-1-P (2 mM) was respectively incubated with 0, 0.09, 0.23, 0.46, 0.91, 1.37 and 1.82 mg (DCW) BL21-TmαGP cells in a 320-μL mixture consisting of 3 mM UTP, 20 mM Mg^2+^and 50 mM sodium phosphate (pH9.0). All experiments were repeated three times. About 4.28 g DCW of StUSP-expressing cells/L reaction mixture was determined to be the most suitable value for UDP-Glc formation.





Fig. S7 Optimization of usage of UTP in UDP-Glc formation by BL21-TmαGP cells. BL21-TmαGP cells (4.3 mg DCW per milliliter))were incubated with the substrate UTP, the concentration of which was varied from 0 to 6 mM, in a mixture containing 2 mM G1P, 20 mM Mg^2+^ and 50 mM sodium phosphate (pH9.0). Bars indicate the range of assay results from three different batches.





Fig. S8 Analysis of PiUDH catalysis in whole-cells by temperature. BL21-PiUDH cells (17.5 mg/ml DCW) were incubated in a mixture consisting of 2.3 mM UDP-Glc, 1.5 mM NAD^+^ and 200 mM sodium phosphate (pH10) at 50, 60, 65, 70, 75, 80, and 85 °C, respectively. The reactions were terminated by chilling on ice for 5 min, and then centrifuged at 12000 g for 20 min at 4 °C, and the supernatants were detected by HPLC. Bars indicate the range of assay results from three different batches.





Fig. S9 pH profile of production of UDP-GlcA by whole cells expressing PiUDH. Reactions were performed in a mixture consisting of 2.3 mM UDP-Glc, 1.5 mM NAD^+^, 200 mM sodium phosphate, and 17.5 mg/ml (DCW) BL21-PiUDH cells at pH values in the range 2.5–13. Bars indicate the range of assay results from three different batches.





Fig. S10 Effect of reaction time on UDP-GlcA formation by whole cells expressing PiUDH. Reactions were performed in a mixture consisting of 2.3 mM UDP-Glc, 1.5 mM NAD^+^, 200 mM sodium phosphate (pH10), and BL21-PiUDH cells (17.5 mg DCW per milliliter), for 0, 10, 20, 30, 40, 60, 90, 120, 150, and 180 min, respectively.





Fig. S11 Optimization of usage of NAD^+^ in whole-cell catalysis by cells expressing PiUDH. BL21-PiUDH cells (17.5 mg DCW per milliliter) were incubated with the cofactor NAD^+^, the concentration of which was varied from 0 to 1.2 mM, in a mixture containing 2.3 mM UDP-Glc and 200 mM sodium phosphate (pH9.0) for 3 h. Bars indicate the range of assay results from three different batches.





Fig. S12 Activity assays of BL21-PiUDH-TkNOX cells by quantity of cells added. Bars indicate the range of assay results from three different batches.
